# A High-Fat Diet Enriched with Low Omega-6 to Omega-3 Fatty Acid Ratio Reduced Fat Cellularity and Plasma Leptin Concentration in *Sprague-Dawley* Rats

**DOI:** 10.1155/2013/757593

**Published:** 2013-10-30

**Authors:** A. W. Tekeleselassie, Y. M. Goh, M. A. Rajion, M. Motshakeri, M. Ebrahimi

**Affiliations:** ^1^Department of Veterinary Preclinical Sciences, Faculty of Veterinary Medicine, Universiti Putra Malaysia (UPM), 43400 Serdang, Selangor, Malaysia; ^2^Institute of Tropical Agriculture, Universiti Putra Malaysia (UPM), 43400 Serdang, Selangor, Malaysia; ^3^Department of Food Science, Faculty of Food Science and Technology, Universiti Putra Malaysia (UPM), 43400 Serdang, Selangor, Malaysia

## Abstract

This study was aimed to investigate the effects of dietary fatty acids on the accretion pattern of major fat pads, inguinal fat cellularity, and their relation with plasma leptin concentration. Forty *Sprague-Dawley* rats were randomly assigned into four groups and received the following diets for 22 weeks: (1) standard rat chow diet (CTRL), (2) CTRL + 10% (w/w) butter (HFAR), (3) CTRL + 3.33% (w/w) menhaden fish oil + 6.67% (w/w) soybean oil (MFAR), and (4) CTRL + 6.67% (w/w) menhaden fish oil + 3.33% (w/w) soybean oil (LFAR). Inguinal fat cellularity and plasma leptin concentration were measured in this study. Results for inguinal fat cellularity showed that the mean adipocyte number for the MFAR (9.2 ∗ 10^5^ ± 3.6) and LFAR (8.5 ∗ 10^5^ ± 5.1) groups was significantly higher (*P* < 0.05) than the rest, while the mean adipocyte diameter of HFAR group was larger (*P* < 0.05) (46.2 ± 2.8) than the rest. The plasma leptin concentration in the HFAR group was higher (*P* < 0.05) (3.22 ± 0.32 ng/mL), than the other groups. The higher inguinal fat cellularity clearly indicated the ability of the polyunsaturated fatty acids (PUFA) and butter supplemented diets to induce hyperplasia and hypertrophy of fat cells, respectively, which caused adipocyte remodeling due to hyperleptinemia.

## 1. Introduction

Obesity is closely linked to a wide array of pathophysiologic consequences including insulin resistance, type 2 diabetes mellitus, hypertension, hyperlipidemia, and atherosclerosis [1]. The epidemic of obesity is increasing in most parts of the world and poses a major challenge to the prevention of chronic noncommunicable diseases [2, 3]. The exact etiology of obesity remains unclear but it appears to be a complex combination of genetic, metabolic, and environmental factors [4]. Dietary patterns characterized by a low intake of vegetables and fruits and a high intake of fatty meat have been identified as a risk factor for obesity [5]. The major change in obesity of the body is clearly the increase in the amount of adipose tissue [6, 7]. Both the amount and dietary fat type are important in regulating fat pad weight through hyperplasia and/or hypertrophy [8]. Adipose tissue cell size has been proposed to modulate the risk of obesity-related disorders [9–11]. Currently, it is apparent that adipocytes are not simply a storage reservoir of fat but are active endocrine organs that secrete a large number of proteins termed adipokines which are involved in the control of several metabolic functions [12, 13]. An increase in fat mass leads to a dysregulation of circulating adipokine levels and relatively causes a pathogenic effect associated with obesity by triggering insulin resistance [6]. It has been observed that the adipocyte size is an important determinant for the secretion of several adipokines [10, 14]. Measurement of circulating adipokine levels may serve as a useful biomarker to evaluate obesity associated health problems [6]. Adipocytokines such as TNF-*α*, adiponectin, resistin, and leptin, synthesized and secreted by adipocytes, have been found to be linked to insulin resistance associated with obesity [15, 16]. Among the adipokines, leptin showed a strong correlation with body mass index, fat accumulation, and insulin resistance [17, 18]. Thus the current study was conducted to assess the impact of high fat diets enriched with different ratios of n-6 : n-3 polyunsaturated fatty acids on fat accretion, inguinal fat cellularity, and plasma leptin concentration. 

## 2. Materials and Methods 

### 2.1. Animal and Diets

Forty male *Sprague-Dawley* rats (9 weeks old) with body weight of 300–320 g were caged individually and acclimatized under animal house conditions and received a standard chow diet for two weeks. After random allocation into four groups (*n* = 10), they were provided the treatment diets. The treatment diets included (1) standard rat chow diet (CTRL), (2) CTRL + 10% (w/w) butter from dairy cow (HFAR), (3) CTRL + 3.33% (w/w) menhaden fish oil + 6.67% (w/w) soybean oil (MFAR), and (4) CTRL + 6.67% (w/w) menhaden fish oil + 3.33% (w/w) soybean oil (LFAR). The chemical constituents of the standard chow diet is presented in Table 1. The n6 : n3 polyunsaturated fatty acid ratio in the CTRL, HFAR, MFAR, and LFAR diets was 154.8, 59.8, 4.8, and 1.6, respectively (Table 2). Rats in the treatment groups received their respective diets for 22 weeks and body weights were recorded weekly. They were provided with clean water *ad libitum* and maintained in a controlled room environment (temperature of 23 ± 1°C, humidity of 85%, and a 12 hr dark and light cycle). All experimental protocols were in accordance with the guidelines of the Institute of Animal Care and Use Committee, Faculty of Veterinary Medicine, Universiti Putra Malaysia.

### 2.2. Body Composition Determination

At the end of the dietary intervention, the body composition and fat accretion pattern of major fat depots were determined using the dissection methods described by [19, 20]. Rats were fasted overnight and blood samples were collected under ketamine and xylasine anesthesia after exposing the abdomen by making a T-line incision. Following acquisition of the blood sample, the rats were sacrificed by decapitation. Skinning was performed by carefully separating the skin from the underlying subcutaneous fat tissue. Inguinal, retroperitoneal, mesenteric, and epididymal fat pads were carefully separated on the basis of their anatomic landmarks.

### 2.3. Lipid Extraction, FAME Preparation, and Gas Liquid Chromatography

Total lipid extraction from the treatment diets was carried out according to Folch et al. [21]. Transmethylation of the extracted fatty acids to fatty acid methyl esters (FAME) was carried out using 14% methanol-boron trifluoride according to methods of AOAC [22]. The FAME was analyzed by Hewlett-Packard Gas-Liquid Chromatography (Hewlett-Packard, Avondale, PA) using Supelco-SP 2330 (30 m, 0.25 mm ID, 0.20 *μ*m film thickness) column (Supelco, Inc. Bellefonte, PA). Ultrapure nitrogen obtained from a nitrogen generator (Dominick Hunter) was used as the carrier gas at 40 mL/min. Ultrapure hydrogen (Dominick Hunter) and compressed air (Pantai Timur Sdn. Bhd) were used in the flame ionization detector. The injector and detector port temperatures were set at 250°C. The column temperature was set at a range of 100–190°C with temperature being programmed at a rate of 7.2°C/min increment to facilitate optimal separation. The identification of individual fatty acids was made by comparing the retention time with the peaks of authentic standards based on their equivalent chain length number [23]. Peak areas were determined using HP-3393A Integrator (Hewlett-Packard, Avondale, PA). Automatic expression of peak areas as the absolute amount of a detected fatty acid was obtained with a programmed PC under Microsoft Excel 2003 (Microsoft Corp., Redmond, USA).

### 2.4. Preparation of Isolated Fat Cells

At the time of necropsy, one gram of inguinal fat was taken. Isolated fat cells were prepared following the method of Rodbell with some modifications [24]. The adipose tissue was minced into small fragments with a pair of scissors and placed in a plastic tube containing phosphate buffer saline (PBS) supplemented with 5 mL of 2 mg/mL of type II collagenase from *Clostridium hemolyticum* (Sigma, Sigma-Aldrich, St. Louis, MO, USA). The tissue was then incubated in a water bath at 37°C for 50 minutes with occasional shaking. At the end of incubation, undigested fibrous tissues were removed using forceps. To stop the effect of collagenase, PBS was added and the suspension was centrifuged at 200 g for five minutes. The infranatant was removed by gentle aspiration using a plastic Pasteur pipette. The latter procedure was repeated twice. Finally PBS was added to bring the suspension to a total volume of 5 mL for determination of the number and diameter of adipocyte cells.

### 2.5. Determination of Inguinal Fat Cellularity

For determination of the adipocyte number, aliquots of the final suspension adipocytes were placed on a Neubauer haemocytometer. Adipocytes were observed under an Olympus microscope BX51 (Olympus, Tokyo, Japan) and photographs were taken using an image analysis software (cc-12 soft imaging system). Adipocyte diameters were also measured by direct Olympus microscopy BX51 (Olympus, Tokyo, Japan) using the image obtained from the previous adipocyte enumeration. The mean adipocyte diameter was calculated from the diameters of 200 cells. 

### 2.6. Plasma Leptin Measurement

Plasma leptin concentration was quantified using a mouse/rat leptin enzyme immunoassay (EIA) kit (SPI-BIO, Massy, France). The leptin concentration of plasma samples was determined from the sample absorbance using a line of best fit equation drawn by the absorbance and concentration of a leptin standard.

### 2.7. Data Analysis

Data sets obtained from body weight, fat accretion pattern, adipocyte cellularity, and plasma leptin concentration were analyzed using an analysis of variance and the results were presented as means ± SEM. When there were significant differences detected, *post hoc* comparisons of means were performed using the least significant difference (LSD). The significance level was set at *P* < 0.05. The SPSS 16 software (SPSS, Chicago, IL, USA) was used for analysis of all data sets.

## 3. Results

### 3.1. Body Weight and Fat Accretion

The body weight of HFAR, MFAR, and LFAR groups was found to be significantly higher (*P* < 0.05) than that of the CTRL group. However, variations among the HFAR, MFAR, and LFAR rats in terms of body weight were not significant. 

With regard to the major fat pad accretion, the HFAR and LFAR fed rats showed a significantly higher amount of epididymal (*P* < 0.05) and retroperitoneal (*P* < 0.01) fat mass compared to their CTRL counterparts (Table 3). In addition, the HFAR rats manifested substantial amounts (17.14 ± 1.40 g) of inguinal fat. In the HFAR group, a significantly (*P* < 0.05) higher weight (34.8 ± 2.8 g) of total subcutaneous fat was also recorded. Although no significant variation was observed in the deposition pattern of the major fat depots among the dietary fat supplemented groups, the LFAR fed rats manifested lower amounts of most of major fat depots compared to the MFAR fed rats. Moreover, the fat to lean mass ratio for LFAR and MFAR rats did not show significant variations unlike HFAR fed rats when compared to the CTRL fed rats.

### 3.2. Dietary Fatty Acid Profile

The fatty acid profile of treatment diets is presented in Table 2. The HFAR diet contained a significantly higher (*P* < 0.05) saturated fatty acid accounting for more than 50% of the total fatty acids. On the other hand, the MFAR diet revealed high amounts of n-3 fatty acids (39.2%) doubling the concentration detected in the LFAR diet (16.1%). However, in the CTRL and HFAR treatment diets, n-3 fatty acids accounted for less than 1% of the total fatty acids. In terms of the total n-6 fatty acids, the LFAR diet took the lead with 39.2% of the total fatty acids followed by 26.4% in MFAR diet. 

### 3.3. Inguinal Fat Cellularity

The adipocyte number and sizes are shown in Table 3. The data clearly indicate that the type of dietary fat had a pronounced effect on fat cell cellularity. The mean adipocyte number per gram of inguinal fat of polyunsaturated fatty acid (PUFA) supplemented groups such as MFAR and LFAR exhibited a significantly higher (*P* < 0.5) count than the other two groups. Despite higher counts, the figures did not reach significant levels. The mean diameter of adipocytes from HFAR group rats was significantly (*P* < 0.05) larger than the rest of the treatment groups. 

### 3.4. Plasma Leptin Concentration

**Figure 1:**
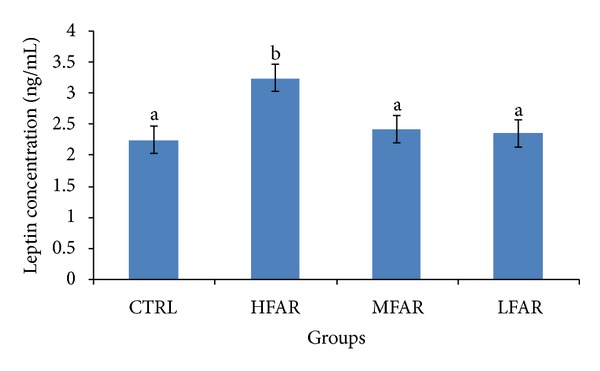
Plasma leptin concentration of rats in the four treatment groups. ^a, b^Different letters among groups indicate significant difference at *P* < 0.05.

Butter supplemented rats (HFAR) acquired a significantly higher (*P* < 0.05) leptin concentration (3.22 ± 0.32 ng/mL) compared to the rest of the groups (Figure 1). However, the plasma leptin concentration of MFAR (2.37 ± 3.2 ng/mL) and LFAR (2.29 ± 0.35 ng/mL) was found to be comparable with the control rats (CTRL) (2.16 ± 0.11 ng/mL).

## 4. Discussion

The present study was carried out to investigate the effects of different ratios of dietary fatty acids on fat accretion, adipocyte cellularity, plasma leptin concentration, and body weight gain. 

Groups supplemented with an additional 10% (w/w) dietary fat showed a significantly higher body weight indicating the role of the additional energy and calorie intake in body weight gain as was also noted by Hynes et al. [25]. Considerable variation was not recorded in terms of the body weight as well as the fat accretion pattern among the dietary fat supplemented groups. In agreement with our findings, it had also been reported that there was absence of significant differences in body weight and fat accretion in rats in response to different dietary PUFA and saturated fatty acid sources [25–27]. Hill et al. [28] also reported that rats fed on different dietary fatty acid supplementation had similar body weights although they noted that there was a differential effect of dietary fatty acids on the fat accretion in different fat pads. The fish oil-fed rats had less total body fat and less intra-abdominal fat than those on diets containing lard or corn oil [28]. 

From the additional 10% fat supplemented groups, only the LFAR group that had a lower n6 : n3 ratio showed a close resemblance to the CTRL group in terms of the total weight. The closeness of LFAR rats to CTRL rats in terms of weight in total was in agreement with Shimomura et al. [29] and Takeuchi et al. [30] who had reported that n-6-rich sunflower oil produced less body fat accumulation than beef-tallow feeding in rats. The authors claimed that the increased diet induced thermogenesis in soybean oil supplemented rats compared to beef tallow supplemented rats was a reason for the observed decrease in fat mass. The HFAR and MFAR rats manifested a high amount of fat accretion in terms of overall total fat, visceral fat, and epididymal and retroperitoneal fat mass in comparison to CTRL rats. In addition, the HFAR rats also exhibited a significantly higher amount of inguinal and overall subcutaneous fat compared to the CTRL rats. 

The types of polyunsaturated fatty acid used, the quantity of fat in the diets, and the length of time studied were among the factors which affect body weight regulation and composition [31, 32]. Different types of fatty acids display different metabolic behaviors such as oxidation and deposition rate that may contribute to a change in body fat and energy metabolism [33, 34]. The long chain derivatives of *α*-linolenic acid (ALA) such as eicosapentaenoic acid and docosahexanoic acid (EPA and DHA) undergo less *β*-oxidation compared to *α*-linoleic acid (LA) [35]. This was reflected in our study where the LFAR rats which consumed more DHA and EPA showed a significantly lower visceral fat mass unlike the MFAR match which was mainly fed n-6 PUFA. The other mechanism that explains how saturated fatty acids (SFA) promote fat deposition was the difference in the stereoisomeric configuration of fat molecules. Stereospecificity of most native oils and fats favors PUFA or monounsaturated fatty acids (MUFA) in the Sn-2 position, whilst SFA were mainly distributed at the Sn-1/3 positions [36]. 

The results of inguinal fat cellularity study revealed the distinct effect of dietary fats on the size and number of adipocytes. It was indicated that the adipocyte diameter was higher in HFAR rats and on the other hand the adipocyte number was higher in the MFAR and LFAR rats. The HFAR rats had a significantly higher inguinal fat mass and this was attributed mainly to the hypertrophy of the existing adipocytes. A similar finding was documented by Cleary et al. [37] regarding the relationships between the type of dietary fats and white adipose tissue development. They reported that inguinal fat hypertrophy and hyperplasia were observed in pups provided with coconut oil and safflower, respectively. Parrish et al. [38] showed that hypertrophy of perirenal and epididymal adipose tissues is lower with high-fat diets enriched in MUFA and PUFA than with high-fat diets enriched with SFA. The differential effect of the fatty acids on cellularity of the inguinal fat pad could be explained by the ability of long chain PUFA to promote hyperplasia through presumably enhanced differentiation of adipose precursor cells whereas long chain SFA promote hypertrophy through enhanced TAG accumulation in differentiated cells [39]. Accumulations of fat stores and fat sizes were known as important functional determinants of the adipocytes themselves. These were central to the postulated influence of obesity on glucose-lipid metabolism in the mammalian body through their adipocytokines.

Plasma leptin quantification showed a significantly higher concentration of leptin in butter supplemented rats (HFAR). In agreement with our findings, a negative association between changes in plasma leptin concentration and dietary n-3 PUFA intake had also been reported by Reseland et al. [40]. Moreover, diets enriched with n-3 fatty acids showed lower plasma leptin levels in rats when compared to the group receiving lard [41]. A similar finding was also reported by Mori et al. [42] in human subjects.

An elevated plasma leptin in the HFAR rats could be linked to the observed hyperinsulinemia in this group of rats. From studies in human and rodents, it appeared that an increase in plasma insulin paralleled an increased plasma leptin concentration [43, 44]. It has been suggested that insulin increases leptin production indirectly via its effects to increase glucose utilization and increased flux of glucose in adipose tissue shown to stimulate the hexosamine pathway and leptin production [45].

Another explanation for the rise in the plasma leptin level of HFAR rats was the relatively higher fat mass, particularly the subcutaneous fat depot. In rodents, the plasma leptin level had been correlated with the percentage of body fat [46]. Changes in adipocyte size have been shown to influence the concentration of adipokines produced by adipocytes. Studies by Skurk et al. [14] in humans and Guo et al. [47] in mice reported that the plasma leptin concentration was positively related to adipocyte size. In agreement with Skurk et al. [14] and Guo et al. [47], the increase in adipocyte size of HFAR rats in our study also paralleled the hyperleptinemia in the rats. 

## 5. Conclusion

Despite the absence of changes in body weight among dietary fat supplemented rats, dietary fat supplementation in the form of butter, soybean, and menhaden fish oil to the control chow diet produced a distinct effect on inguinal fat cellularity and plasma leptin concentration. Adipocytes from MFAR and LFAR rats tended to be hyperplastic, whereas adipocytes from HFAR rats were hypertrophied. In addition, hyperleptinemia was noticed in the HFAR rats unlike MFAR and LFAR rats. We concluded that the distinct metabolic properties of dietary fatty acids on the mode of adipose tissue growth could account for the observed changes.

## Figures and Tables

**Table 1:** Chemical constituents of the standard chow diet (dry-weight basis).

Constituents (per 100 g feed)	Diet
Crude fat (g)	3
Crude protein (g)	20
Carbohydrate (g)	51
Crude fiber (g)	7.3
Ash (g)	9.2
Calcium	0.9
Phosphorus	0.5

**Table 2:** Plasma fatty acid profile of treatment groups.

Fatty acid (g/100 ml)	Experimental diets				
Name	CTRL	HFAR	MFAR	LFAR	SEM
Capric acid (10:0)	n/d	n/d	0.5^a^	0.5^a^	0.14
Lauric acid (12:0)	n/d	0.3^a^	0.2^a^	0.3^a^	0.07
Myristic acid (14:0)	0.5^a^	0.7^ab^	0.7^bc^	1.0^c^	0.10
Pentadecanoic acid (15:0)	0.6^ab^	0.5^a^	0.7^ab^	0.8^b^	0.06
Palmitic acid (16:0)	18.1^ab^	19.9^b^	16.6^a^	19.0^ab^	0.70
Palmitoleic acid (16:1)	0.7^a^	0.5^a^	1.2^b^	1.2^b^	0.18
Heptadecanoic acid (17:0)	0.6^a^	0.5^a^	0.7^ab^	0.9^b^	0.09
Stearic acid (18:0)	11.7^ab^	12.4^b^	11.2^a^	10.4^a^	0.42
Oleic acid (18:1)	9.9^a^	12.3^a^	13.2^a^	11.2^a^	0.71
Linoleic acid (18:2(n-6))	22.3^a^	19.3^a^	23.8^a^	24.3^a^	1.13
Linolenic acid (18:3(n-3))	0.4	n/d	n/d	0.01	0.01
Arachidic acid (20:0)	0.8^b^	0.4^a^	0.7^b^	0.9^b^	0.11
Cis-11-Eicosenoic (20:1)	0.4^a^	0.6^ab^	0.4^a^	0.8^b^	0.10
Cis-11,14,17-Eicosatrienoic (20:3)	0.5^a^	0.5^ab^	0.7^b^	n/d	0.15
Arachidonic acid (20:4(n-6))	30.9^b^	28.9^b^	20.1^a^	14.6^a^	1.81
Eicosapentaenoic acid (20:5(n-3))	n/d	n/d	2.5^a^	5.4^b^	1.28
Behenic acid (22:0)	0.8^a^	1.5^b^	1.6^ab^	1.5^ab^	0.18
Docosapentaenoic acid (22:5(n-3))	n/d	n/d	0.6^a^	1.1^b^	0.27
Docosahexanoic acid (22:6(n-3))	2.0^a^	1.8^a^	4.6^b^	6.4^b^	0.10
Total saturated fatty acids^ns^	33.0	36.2	33.0	35.3	0.82
Total unsaturated fatty acids^ns^	67.0	63.8	67.0	64.7	0.82
Total monounsaturated fatty acids^ns^	11.0	13.4	14.7	13.2	0.79
Total PUFA n-3	2.4^a^	1.8^a^	7.7^b^	12.91^c^	0.60
Total PUFA n-6	53.2^b^	48.7^b^	43.9^ab^	38.9^a^	3.05
n-6 : n-3 ratio	22.17^b^	34.1^b^	5.7^a^	3.02^a^	1.91
U : S ratio^ns^	2.0	1.8	2.04	1.8	0.07
P : S ratio	1.7^c^	1.4^a^	1.6^bc^	1.5^ab^	0.06

^a,b,c^Values with different superscripts within rows are significantly different (*P* < 0.05); n/d: not detected; S: saturated; U: unsaturated; P: polyunsaturated; ^ns^not significant. SEM:  standard error of means.

CTRL:  control group; HFAR:  high n-6 : n-3 fatty acid ratio group; MFAR:  medium n-6 : n-3 fatty acid ratio group; LFAR: low n-6 : n-3 fatty acid ratio group.

**Table 3:** Body composition, fat accretion, and inguinal fat cellularity of rats (Mean ± SEM).

Treatment groups	CTRL	HFAR	MFAR	LFAR
Live weight (g)	456.9 ± 10.6^a^	510.6 ± 13.2^b^	511.8 ± 15.0^b^	502.50 ± 17.0^b^
Actual carcass Weight (g)	291.6 ± 8.6^a^	333.8 ± 8.7^b^	330.1 ± 10.4^b^	311.5 ± 11.3^ab^
Total muscle (g)	187.1 ± 5.1^a^	207.5 ± 5.6^b^	208.2 ± 5.6^b^	195.8 ± 7.9^ab^
Inguinal fat (g)	10.64 ± 1.29^a^	17.14 ± 1.40^b^	13.98 ± .98^ab^	14.12 ± 1.38^ab^
Retroperitoneal fat (g)	6.67 ± 1.05^a^	12.16 ± 0.90^b^	10.67 ± 1.02^b^	9.51 ± 1.02^ab^
Mesenteric fat (g)	4.46 ± 0.49^a^	6.31 ± 0.51^b^	6.89 ± 0.64^b^	6.61 ± 0.42^b^
Epididymal fat (g)	5.67 ± 0.61^a^	7.99 ± 0.72^b^	7.71 ± .77^b^	6.33 ± 0.49^ab^
Anterior fat (g)	12.9 ± 1.6^a^	17.8 ± 1.5^b^	16.1 ± 1.0^ab^	14.9 ± 0.9^ab^
Total visceral fat (g)	18.4 ± 2.3^a^	29.0 ± 2.0^b^	27.6 ± 2.5^b^	24.4 ± 1.8^ab^
Total subcutaneous fat (g)	23.5 ± 2.9^a^	34.8 ± 2.8^b^	30.1 ± 1.8^ab^	29 ± 2.2^ab^
Total fat (g)	41.9 ± 5.0^a^	63.8 ± 4.7^b^	57.7 ± 4.0^b^	53.4 ± 3.9^ab^
Fat to lean mass ratio	0.22 ± 0.04^a^	0.31 ± 0.02^b^	0.28 ± 0.02^ab^	0.27 ± 0.02^ab^
Adipocyte number ∗ 10^5^/g	7.2 ± 3.3^a^	6.7 ± 4.6^a^	9.2 ± 3.6^b^	8.5 ± 5.1^b^
Adipocyte diameter ∗ 10^6^ *µ*m/g	30.6 ± 1^a^	46.2 ± 2.8^b^	35.1 ± 3.8^a^	37.3 ± 1.8^a^

^a,b^Values with different superscripts within rows are significantly different at *P* < 0.05. CTRL: Control group; HFAR: High n-6: n-3 fatty acid ratio group; MFAR: Medium n-6: n-3 fatty acid ratio group; LFAR: Low n-6: n-3 fatty acid ratio group.
